# Insights into the Structural Modification of Selenium-Doped Derivatives with Narrowband Emissions: A Theory Study

**DOI:** 10.3390/molecules29194589

**Published:** 2024-09-27

**Authors:** Qing Zhang, Tao Liu, Xin Huang, Kunyan Wang, Fangxiang Sun, Xin Wang, Chunyan Lv

**Affiliations:** 1 Department of Materials Chemistry, Huzhou University, Huzhou 313000, China; 2 Henan-Macquarie University Joint Centre for Biomedical Innovation, Henan Key Laboratory of Brain Targeted Bio-Nanomedicine, School of Life Sciences, Henan University, Kaifeng 475004, China

**Keywords:** narrowband emission, selenium-doped, structural modification, pressure effects, TD-DFT, ONIOM (QM/MM) calculations

## Abstract

The research on boron/nitrogen (B/N)-based multiresonance thermally activated delayed fluorescence (MR-TADF) emitters has been a prominent topic due to their narrowband emission and high luminous efficiency. However, devices derived from the common types of narrowband TADF materials often experience an efficiency roll-off, which could be ascribed to their relatively slow triplet–singlet exciton interconversion. Since inserting the heavy Se atom into the B/N scheme has been a proven strategy to address the abovementioned issues, herein, extensive density functional theory (DFT) and time-dependent DFT (TD-DFT) simulations have been employed to explore the effects of the structural modification on a series of structurally modified selenium-doped derivatives. Furthermore, the two-layered ONIOM (QM/MM) model has been employed to study the pressure effects on the crystal structure and photophysical properties of the pristine CzBSe. The theoretical results found that the introduced tert-butyl units in Cz-BSeN could result in a shorter charge transfer distance and smaller reorganization energy than the parent CzBSe. In contrast to directly incorporating the *o*-carborane (*Cb*) unit to CzBSe, incorporating the bridged phenyl units is important in order to achieve narrowband emissions and high luminous efficiency. The lowest three triplet excited states of CzBSe, Cz-BSeN and PhCb-BSeN all contribute to their triplet–singlet exciton conversions, resulting in a high utilization of triplet excitons. The pressure has an evident influence on the photophysical properties of the aggregated CzBSe and is favored for obtaining narrowband emissions. Our work is promised to provide a feasible strategy for designing selenium-doped derivatives with narrowband emissions and rapid triplet–singlet exciton interconversions.

## 1. Introduction

Originating from the conception by Adachi and coworkers in 2012 [1], purely organic thermally activated delayed fluorescence (TADF) materials have garnered increasing research attention in organic light-emitting diodes (OLEDs) [2,3,4]. By virtue of the reverse intersystem crossing (RISC) process triggered by the small energy gaps (Δ*E*_ST_) between the singlet excited state (S_1_) and relevant triplet excited states, purely organic TADF emitters can also take advantage of triplet excitons for luminescence without using rare noble metals [5,6,7]. Although the intramolecular charge-transfer (ICT) feature of the traditional donor–acceptor (D-A)-type TADF emitters could result in a sufficiently small Δ*E*_ST_, it is inevitably accompanied by broad emission spectra with an FWHM greater than 70 nm, making it challenging to realize an ultrahigh definition (UHD) display with a high color purity [8,9]. In addition to the spectral broadening, the ICT properties are usually associated with a weaker emission oscillator strength (*f*), since *f* is proportional to the overlap of holes and electrons.

To address the shortcomings of D-A-type TADF emitters, in 2016, Hatakeyama and co-workers proposed a boron/nitrogen (B/N)-based method to construct purely organic multiresonance TADF (MR-TADF) emitters with narrowband emissions and a high luminous efficiency [10]. In contrast to fluorescent organic light-emitting materials with narrowband emissions, MR-TADF emitters could also use triplet excitons. Although some cyclometalated complex-based phosphorescent materials could exhibit a high color purity, the use of rare noble metals increases its manufacturing cost. The short-charge transfer (SR-CT) characteristic arising from the alternately distributed holes and electrons enables both the low Δ*E*_ST_ and considerable luminous efficiency of MR-TADF emitters. Meanwhile, the rigid and planar natures of the MR skeleton can reduce structural relaxations of the excited state, which is in favor of narrowband emissions. Consequently, a small Δ*E*_ST_, intense luminous efficiency, and extremely sharp emissions can be achieved in an MR-TADF emitter. Motivated by the above advantages, B/N-based narrowband emitters have gained increasing attention and full-color emissions with extremely narrow spectral features covering the whole visual region have been realized [11,12,13]. Despite the promising features of those emitters, nevertheless, the corresponding devices typically suffer from a serious efficiency roll-off at high brightness, which could be attributed to the serious triplet–triplet annihilation (TTA) and triplet–polaron annihilation (TPA) arising from the small reverse intersystem crossing rate constant (*k*_RISC_) [14,15,16].

**Figure 1:**
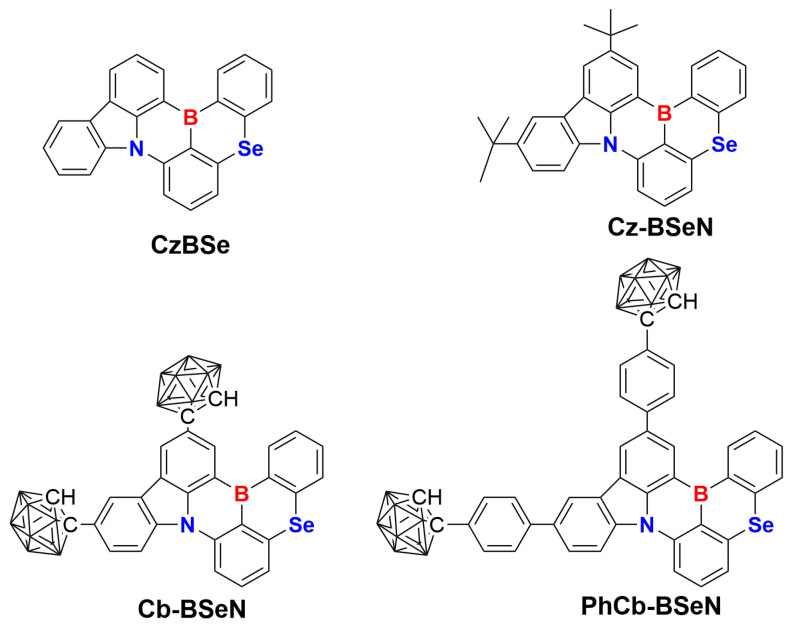
Chemical structure of the studied Se-doped derivatives, in which CzBSe and Cz-BSeN have been reported in the experiment, and Cb-BSeN and PhCb-BSeN were designed in the theory.

In 2022, Yasuda and coworkers developed the first selenium (Se)-doped TADF emitter, namely, CzBSe, exhibiting a record-high *k*_RISC_ over 1 × 10^8^ s^−1^ with a narrow emission FWHM of 33 nm [17]. In the same year, another two Se-doped narrowband emitters were reported by introducing tert-butyl units (Cz-BSeN) and 3,6-ditertbutylcarbazole moieties (DCz-BSeN) *para*-positioned at the N atom of the CzBSe [18]. As expected, both emitters exhibit a fast *k*_RISC_ of 7.5–8.8 × 10^6^ s^−1^ and the roll-offs of their corresponding devices are much lower than the pristine device without incorporating a heavy Se atom [19]. In addition, inserting the heavy Se atom has been a proven strategy for achieving a high utilization of triplet excitons with large *k*_RISC_ [20,21,22,23]. *O*-carborane (*Cb*), an icosahedral boron cluster showing a three-dimensional pseudo-cage structure, has been demonstrated to be a prospective building block for photofunctional materials [24,25,26,27,28]. Inspired by those, two structurally modified Se-doped derivatives were designed in theory by incorporating the spherical *o*-carborane (Cb-BSeN), as well as the bridged phenyl and *o*-carborane units (PhCb-BSeN) *para*-located at the N atom in the parent CzBSe as bulky peripherals (Figure 1). Following that, the density functional (DFT) and time-dependent DFT (TD-DFT) approaches were adopted to explore the effects of the structure modification on the structural vibrations, narrowband emissions, and charge transfer natures of the four selenium-doped derivatives. Furthermore, the ONIOM (QM/MM) calculations were used to investigate the influence of pressure on the crystal structures and photophysical properties of the experimentally developed CzBSe in the aggregated state.

## 2. Results and Discussion

### 2.1. Nature of Low-Lying Excited States

**Figure 2:**
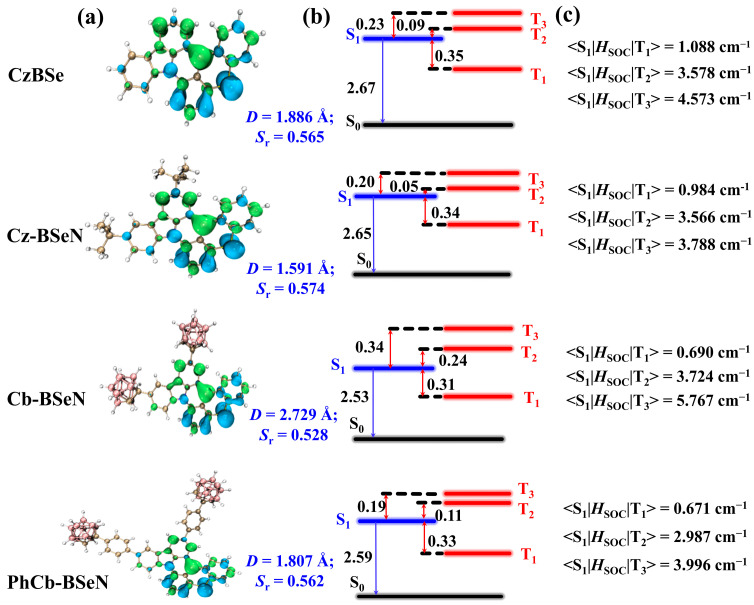
Simulated hole–electron distributions (isosurface value = 0.002) for S_1_→S_0_ transitions; blue and green means hole and electron distributions, respectively (**a**). Predicted vertical excitation energies of S_1_, energy gaps (**b**), and SOC constants (**c**) between S_1_ and its energetically similar triplet states, in which energies are in eV.

To deeply understand the nature of S_1_, the hole and electron distributions of the S_1_→S_0_ transitions were predicted for all the considered systems, as shown in Figure 2a. Moreover, the distance between the centroids of holes and electrons (*D* index) and their overlap degrees (*S*_r_ index) were quantitatively predicted and summarized in Figure 2a. Note that the process of the electronic excitation of S_1_→S_0_ transitions can be described as “hole → electron”. From Figure 2a, the holes and electrons of all the studied molecules are alternately distributed on different atoms, showing MR effects. In detail, holes are located on the N and Se atoms and the *ortho*- and *para*-located C atoms relative to them, while electrons are confined within the electron-deficient B and its *ortho*- and *para*-located C atoms. Note that the Se also contributes to the hole distributions of CzBSe and Cz-BSeN, indicating that the incorporated heavy atom Se could enrich the MR scheme of the B/N-based narrowband emitters. The quantitatively characterized *D* index of 1.886 and 1.591 Å for CzBSe and Cz-BSeN, respectively, are comparable to or even less than the C–S bond length (1.785 Å), showing that short-range CT features can be found in the S_1_ of those two reported MR-TADF emitters. The predicted *S*_r_ index of CzBSe and Cz-BSeN are 0.565 and 0.574 (the theoretical upper limit is 1.0), suggesting that over half of the holes and electrons overlap completely. Therefore, it can be concluded that the alternatingly distributed holes and electrons enable the reported CzBSe and Cz-BSeN with both short-range CT characters and a sufficient overlap of holes and electrons, which is of great importance in obtaining a small Δ*E*_ST_, narrowband emissions, and high luminous efficiency in a molecule. In addition, the smaller *D* index of Cz-BSeN fits well with its narrower emission spectral FWHM (FWHM = 30 nm) compared to that of CzBSe (FWHM = 33 nm). When the spherical *Cb* groups were introduced to the pristine CzBSe, the *D* index of the yielding Cb-BSeN significantly increased to 2.729 Å. The sharply increased *D* index suggests that introducing *Cb* units is unfavorable for achieving shorter charge transfer distance. It is worth noting that the *D* index of PhCb-BSeN, taking phenyl as the bridge to connect the original CzBSe and the *Cb* units, was simulated to be 1.807 Å, which is much smaller than Cb-BSeN (2.729 Å) that obtained by directly incorporating *Cb* moieties to the *para* position of the N atom in CzBSe. From the atomic contribution to holes and electrons summarized in Table S1, it is found that the bridged phenyl units in PhCb-BSeN could affect the atomic contributions of holes and electrons compared to that in Cb-BSeN, especially the contributions of Se atoms, resulting in a more confined hole–electron distribution in PhCb-BSeN and, therefore, a smaller *D* index. In addition, the simulated *S*_r_ index of PhCb-BSeN was 0.562, which is comparable with the pristine CzBSe (0.565) and slightly larger than that of Cb-BSeN (0.528). These results suggest that incorporating the bridged phenyl units is crucial for shortening the hole–electron charge transfer distance and improving their overlap. Overall, the shorter charge transfer distances associated with the larger overlaps of holes and electrons enable the structurally modified PhCb-BSeN promising to show smaller emission spectral FWHMs and higher luminous efficiencies than its pristine CzBSe.

The B3LYP-simulated vertical excitation energies of S_1_ were 2.53 and 2.59 eV for the structurally modified Cb-BSeN and PhCb-BSeN, respectively, and their corresponding maximum wavelengths of emission were 490 and 478 nm, indicating that the introduced *Cb* and phenyl units could redshift the emission of the parent CzBSe (477 nm). The predicted Δ*E*_S1T1_ values were 0.31 eV for Cb-BSeN and 0.33 eV for PhCb-BSeN, which are comparable with those for the reported CzBSe (0.35 eV) and Cz-BSeN (0.34 eV). Note that the predicted vertical energies of T_2_ for CzBSe and Cz-BSeN are only slightly higher than those of S_1_ with the Δ*E*_S1T2_ values of 0.09 and 0.05 eV, respectively. The simulated SOC constants between S_1_ and T_2_ (<S_1_|*H*_SOC_|T_2_>) were 3.578 cm^−1^ for CzBSe and 3.566 cm^−1^ for Cz-BSeN, which are obviously higher than their corresponding <S_1_|*H*_SOC_|T_1_> values of 1.088 cm^−1^ for CzBSe and 0.984 cm^−1^ for Cz-BSeN. Notably, the <S_1_|*H*_SOC_|T_3_> values of 4.573 and 3.788 cm^−1^ predicted for CzBSe and Cz-BSeN are the most significant SOC constants predicted between their S_1_ and the lowest three triplet states. Therefore, the energetically similar S_1_ and T_2_/T_3_ combined with the relatively enhanced <S_1_|*H*_SOC_|T_2_> and <S_1_|*H*_SOC_|T_3_> values suggest that the T_2_ and T_3_ of CzBSe and Cz-BSeN emitters could contribute to their exciton interconversions between the singlet and triplet excited states. As a result, the experimentally reported CzBSe and Cz-BSeN could undergo rapid reverse intersystem crossing processes and the high utilization of triplet excitons. The Δ*E*_S1T2_ values for the designed PhCb-BSeN was 0.11 eV, similar to the 0.09 eV simulated for CzBSe, and its corresponding <S_1_|*H*_SOC_|T_2_> values were predicted to be 2.987 cm^−1^, slightly smaller than the 3.578 cm^−1^ simulated for CzBSe. The T_3_ energy of PhCb-BSeN is somewhat higher than its S_1_ energy with an energy gap of 0.19 eV, which is smaller than the 0.23 eV predicted for the original CzBSe. The <S_1_|*H*_SOC_|T_3_> value of PhCb-BSeN is also the largest SOC constant predicted between S_1_ and its lowest three triplet states. Therefore, similar to the pristine CzBSe, the energetically close-lying S_1_ and T_2_/T_3_ together with the enhanced <S_1_|*H*_SOC_|T_2_> and <S_1_|*H*_SOC_|T_3_> values suggest that the structurally modified PhCb-BSeN is promising for undergoing an effective triplet–singlet exciton interconversion. For comparison, the Δ*E*_S1T2_ and Δ*E*_S1T3_ values of the structurally modified Cb-BSeN were 0.24 and 0.34 eV, respectively, much greater than the corresponding energy gaps simulated for PhCb-BSeN. From the aforementioned analyses, we could envision that the structurally modified PhCb-BSeN has a hope of showing narrower emission spectral FWHMs, higher luminous efficiencies, and a comparable triplet–singlet exciton interconversion, as compared with those of the pristine CzBSe.

### 2.2. Structural Changes and Vibronic Analyses

**Figure 3:**
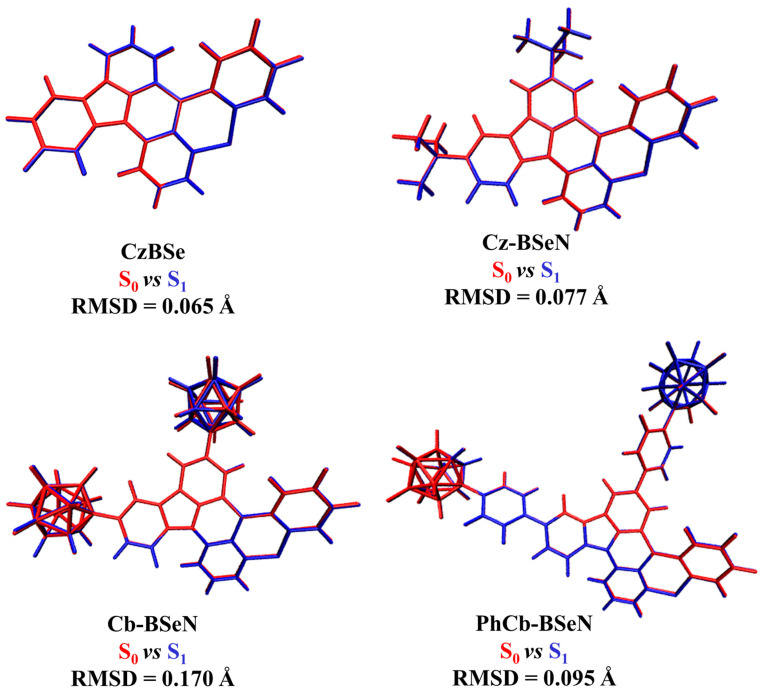
Structural comparisons and simulated RMSD values between S_0_ (red) and S_1_ (blue) for all the studied molecules.

The geometrical relaxation of S_1_ is strongly correlated with its photophysical properties. Herein, the root mean square displacement (RMSD) value, a widely used parameter to compare the global geometrical differences between various structures, was quantitatively simulated to evaluate the structural changes between S_1_ and S_0_. The structural differences and RMSD values of all the considered compounds are summarized in Figure 3. The simulated RMSD value for Cz-BSeN was 0.077 Å, slightly larger than the 0.065 Å predicted for CzBSe. Originating from the obvious structural variations of the incorporated *Cb* units, the RMSD value between S_1_ and S_0_ for the designed Cb-BSeN significantly increases to 0.170 Å, suggesting that the emission of Cb-BSeN is inevitably accompanied by an apparent non-luminescent structural relaxation. However, it is worth noting that, when phenyl groups were used to bridge the spherical *Cb* unit and the pristine CzBSe, the structural changes between S_1_ and S_0_ of the introduced *Cb* units are noticeably suppressed, resulting in the yielding PhCb-BSeN an obviously reduced RMSD value of 0.095 Å. Those results indicate that directly incorporating bulky *Cb* as peripheral units is unhelpful in inhibiting the geometrical changes during emission, while the linked phenyl moiety is particularly important for suppressing the S_1_→S_0_ structural variations of *Cb* units in PhCb-BSeN.

**Figure 4:**
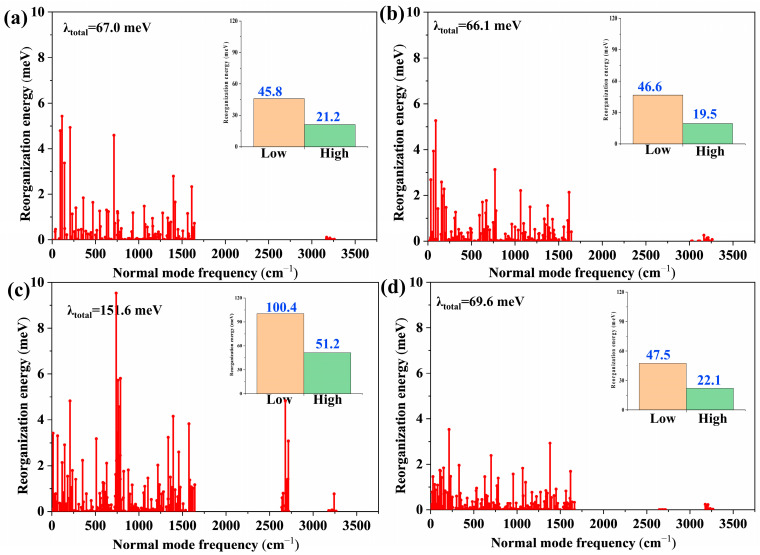
Predicted reorganization energies and corresponding normal mode frequencies between S_0_ and S_1_ for CzBSe (**a**), Cz-BSeN (**b**), Cb-BSeN (**c**), and PhCb-BSeN (**d**), respectively. The inset shows contributions of low- (<1000 cm^−1^) and high-frequency vibrations (≥1000 cm^−1^) to the reorganization energies of all the studied systems.

Generally, the emission spectral FWHM highly depends on the reorganization energy (λ) between S_1_ and S_0_ [29,30,31]. As shown in Figure 4, the simulated reorganization energies were 67.0 and 66.1 meV, respectively, for the reported CzBSe and Cz-BSeN. The slightly smaller reorganization energy of Cz-BSeN coincides well with its experimentally obtained narrower emission spectral FWHM of 30 nm relative to that of 33 nm for CzBSe [17], suggesting that the simulated reorganization energies could well assess the emission spectral FWHM of the investigated emitters. From the insert in Figure 4, the low-frequency vibrations (<1000 cm^−1^) dominate the energy reorganization of all the considered compounds. When introducing the spherical *Cb* units directly to CzBSe, the contribution of both low- and high-frequency vibrations (≥1000 cm^−1^) of the generated Cb-BSeN increased sharply. Compared with Cb-BSeN, incorporating phenyl units could suppress both low- and high-frequency vibrations of the yielding PhCb-BSeN. The predicted reorganization energy for Cb-BSeN was 151.6 meV, which is the largest of all systems considered here. This result shows that incorporating the spherical *Cb* groups directly to the pristine CzBSe as peripherals is unfavorable for achieving a narrower emission spectral FWHM. The largest reorganization energy of Cb-BSeN could be attributed to its considerably larger structural differences between S_1_ and S_0_, which is dominated by the obvious variations of the introduced *Cb* moieties between the two states (Figure 3). Furthermore, it is found from Figure 4 and Figure S1 that the structural vibrations of the low-frequency scissoring and high-frequency stretching of *Cb* units contribute a lot to the reorganization energy of Cb-BSeN, and those vibrations are almost wholly restrained in PhCb-BSeN. As a result, the reorganization energy of PhCb-BSeN, which was designed by inserting a phenyl unit as the bridge to link CzBSe and the *Cb* units, greatly decreased to 69.6 meV. The sharply decreased reorganization energy of PhCb-BSeN suggests that incorporating the linked phenyl group is of particular importance to suppressing the structural relaxation of *Cb* units and, therefore, is favored for obtaining narrowband emissions.

### 2.3. Pressure Effects on Photophysical Properties

**Figure 5:**
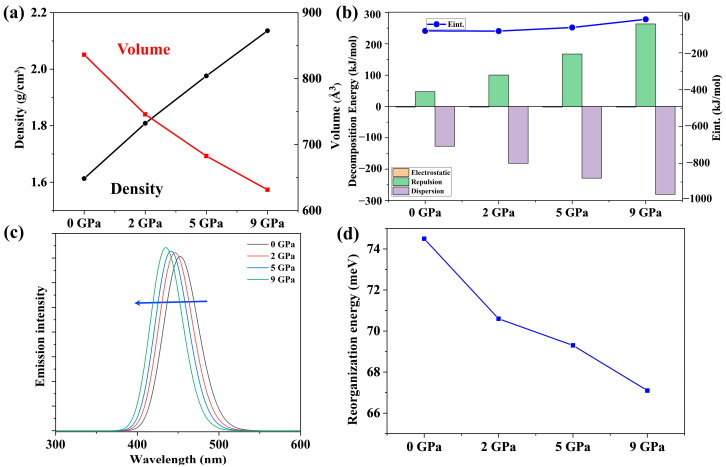
Simulated packing volume and packing density (**a**), EDA-FF-predicted decomposed interactions of the selected π-stacked dimers (**b**), B3LYP-predicted maximum emission peaks (**c**), and the modeled organization energies (**d**) of the aggregated CzBSe at different pressures of 0, 2, 5, and 9 GPa, where the ambient pressure is set as 0 GPa.

Originating from the crystal structure obtained at ambient pressure in the experiment, the photophysical properties of CzBSe under various pressures have been studied. As shown in Figure 5a, the packing densities of CzBSe grow monotonically from 1.61 cm^−3^ at ambient pressure to 2.14 cm^−3^ under 9 GPa, and the packing volumes decrease accordingly upon compression. As depicted in Figure S2, the unit cell of CzBSe is characterized by obvious π…π stacked dimers. Therefore, the π…π stacked dimers were selected as representations to gain insight into the effects of various external pressures on the intermolecular interactions of CzBSe in the aggregated state. Within the energy decomposition analysis scheme based on forcefield (EDA-FF), the total intermolecular interactions can be decomposed into electrostatic, repulsion, and attractive dispersion interactions, respectively. The EDA-FF-predicted decomposed interaction energies are plotted in part b of Figure 5, and the detailed values are given in Table S2 in the Supporting Information. From Figure 5b, the electrostatic interactions are negligible to the intermolecular interactions of the π-stacked dimers, while the repulsion and dispersion interactions dominate the interaction energies. In detail, both the repulsion and dispersion interactions gradually increase, which could be attributed to the decreased intermolecular distance within the monomers of the π-stacked dimers upon compression. The abovementioned analysis indicates that the applied external pressure noticeably influences the crystal structure of CzBSe, therefore affecting its photophysical properties in the aggregated state.

The maximum emission peak of CzBSe simulated at ambient pressure was 453 nm, exhibiting a blue shift relative to the emission obtained in the toluene solution. With applying external pressures, the maximum emission of CzBSe further blue-shifted to 435 nm as the pressure increases from 2 to 9 GPa. The phenomenon suggests that increasing the external pressures is expected to blue-shift the maximum emission of CzBSe in the aggregated state. The reorganization energies of CzBSe at different pressures were simulated to reveal the pressure effects on the emission spectral FWHM in the aggregated state. From Figure 5d, the reorganization energy was predicted to be 74.5 meV at ambient pressure and gradually decreased to 67.1 meV upon compression. Since the emission spectral FWHM is directly proportional to the reorganization energy, the monotonically decreasing reorganization energy suggests that the applied external pressures are beneficial for achieving narrowband emissions, and, with increasing pressures, the emission spectral FWHMs become narrower.

## 3. Methods

### 3.1. Calculations for Solvated Molecule

The geometries of the ground state (S_0_), S_1_, and T_1_ were optimized at the B3LYP/def2SVP level, and all optimizations were confirmed to be stable structures with no imaginary frequencies at the same simulating level of optimization. Since the excitation energies of S_1_ and T_1_ are strongly related to the percentages of HF exchange (HF%) of the adopted hybrid functionals, four functionals with different HF%, B3LYP (20%), PBE0 (25%), MN15 (44%), and M06-2X (56%), have been tested based on the optimized S_1_ and T_1_ structures to select a more accurate approach to predict the vertical excitation energies of the reported CzBSe and Cz-BSeN. Note that the solvation model based on density (SMD) [32] was used to take the solvent effects of the toluene used experimentally into account for the simulated geometries and energies. Table S3 summarizes the predicted vertical energies and energy gaps between S_1_ and T_1_ (Δ*E*_S1T1_). In all four tested hybrid functionals, it is found that the B3LYP-simulated excitation energies for CzBSe and Cz-BSeN are 2.67 (465 nm) and 2.65 eV (467 nm), respectively, which coincidence well with their corresponding 2.60 (477 nm) and 2.59 eV (479 nm) measured in the experiment. Those analyses show that the properties of S_1_ for the considered compounds could be well-described at the B3LYP/def2-SVP level. However, owing to the underestimation of T_1_ energies, the B3LYP-simulated Δ*E*_S1T1_ values of 0.35 and 0.34 eV for CzBSe and Cz-BSeN are much larger than the experimental results of 0.12 and 0.15 eV, respectively. Since the Δ*E*_S1T1_ is of great importance to characterizing TADF properties, the DLPNO-STEOM-CCSD method [33,34,35,36] with the def2-TZVP basis set, a highly correlated wavefunction-based calculation with high accuracy, was employed as the benchmark to value the reliability of the Δ*E*_S1T1_ predicted at the B3LYP/def2-SVP level. The Δ*E*_S1T1_ values simulated by the wavefunction-based calculations are 0.20 eV for CzBSe and 0.19 eV for Cz-BSeN, showing good agreement with their experimental measurements of 0.12 and 0.15 eV. Note that the relative Δ*E*_S1T1_ values of CzBSe and Cz-BSeN simulated at the high-precision DLPNO-STEOM-CCSD/def2-TZVP level agrees with those obtained at the simulating level of B3LYP/def2-SVP. In addition, the B3LYP has been a proven functional to simulate the excitation energies of singlet and triplet states of the B/N-based narrowband emitters [37,38]. Therefore, the Δ*E*_S1T1_ values of all the considered systems herein were qualitatively predicted at the B3LYP/def2-SVP level, since all attempts to simulate the Δ*E*_S1T1_ values of the theoretically designed molecules using the high-precision DLPNO-STEOM-CCSD method have failed.

### 3.2. Crystal Structure Optimization and ONIOM Simulation

Based on the crystal obtained experimentally at ambient pressure, the unit cell of CzBSe at different external pressures was fully optimized by employing the Perdew–Burke–Ernzerhof (PBE) functional [39], implemented in the CASTEP module of the Materials Studio 2020 software [40]. During optimization, the *k*-points were set to 5 × 7 × 5, and both the lattice parameters and atomic locations of the unit cells were completely relaxed. The external pressures were set to 2, 5, and 9 GPa, respectively. Moreover, the experimental crystal obtained at ambient condition was treated as the reference pressure (0 GPa). Starting from the optimized crystal structures abovementioned, the photophysical properties of the reported CzBSe at various pressures were simulated using the two-layered ONIOM model with the hybrid quantum mechanics and molecular mechanics (QM/MM) method [41]. The two-layered QM/MM model of CzBSe is given in Figure S3. In the QM/MM simulation, the centered molecule is free to move and be excited and simulated at the high-precision B3LYP/def2-SVP level. In contrast, the rest molecules are frozen as the solid environment and modeled by the low-level universal force field (UFF) force [42] with the charge equilibration method [43]. The electronic embedding scheme was adopted in all QM/MM simulations to better characterize the electrostatic interactions between the two regions.

All QM/MM and solvated molecule calculations were obtained with the Gaussian 16 software [44], and the wavefunction-based calculations were performed using the ORCA 5.0 program [45]. Distributions of holes and electrons associated with the *D* index (the distance between hole and electron centroids) and *S*_r_ index (the overlapping degree of hole and electron) of those distributions were predicted to gain insights into the nature of electron excitations. The intermolecular interactions of the π-stacked dimer of CzBSe were predicted by employing the energy decomposition analysis (EDA) with the classical molecular force field [46,47,48]. All the aforementioned analyses were performed by using the Multiwfn 3.8 software [49]. The spin-orbit coupling (SOC) constants between S_1_ and the relevant triplet excited states were simulated by employing the Dalton 2020.1 software [50]. Furthermore, the reorganization energy between S_1_ and S_0_ and the normal mode analyses were performed at the optimized S_1_ geometries by using the Dushin program implemented in MOMAP 2020A software [51].

## 4. Conclusions

This study explores the effects of the structure modification on the charge transfer natures, geometrical vibrations, and narrowband emissions of four selenium-doped derivatives. Additionally, the pressure effects on the crystal structure and photophysical properties of the pristine CzBSe have been investigated. Our current results indicate that the incorporated tert-butyl units in Cz-BSeN could result in a shorter charge transfer distance and smaller reorganization energy, which accounts for its smaller emission spectral FWHM than that of CzBSe. In contrast to directly introducing *Cb* moieties to the pristine CzBSe (Cb-BSeN), a combination of *Cb* and phenyl groups (PhCb-BSeN), where phenyl acts as the bridge to connect *Cb* groups to the pristine CzBSe, is beneficial for shortening the charge transfer distance between holes and electrons and enhancing their overlaps. In addition, the incorporated phenyl units could suppress the structural relaxing and vibrations of the *Cb* moieties, giving PhCb-BSeN a considerable decreased reorganization energy relative to Cb-BSeN. The shorter charge transfer distance, and enhanced overlaps between holes and electrons enable PhCb-BSeN hopefully to exhibit both narrowband emissions and high luminous efficiency. The PhCb-BSeN is also expected to display comparable reverse intersystem crossing processes and a high utilization of triplet excitons with the pristine CzBSe. The external pressure has an evident influence on the crystal structure of CzBSe, which consequently affects its photophysical properties in its aggregated state, and the external pressure is favored for obtaining narrowband emission. Our study will likely give ideas for the further research and development of new selenium-doped emitters with narrowband emissions.

## Data Availability

The data are contained within the article or Supplementary Materials.

## Supplementary Materials

The following supporting information can be downloaded at: https://www.mdpi.com/article/10.3390/molecules29194589/s1, Table S1: Atomic contributions to holes and electrons; Table S2: Decomposed interaction energies of the π-stacked dimer; Table S3: Comparison of theoretically and experimentally obtained excited energies; Table S4: Simulated energy gaps by the wavefunction-based STEOM-DLPNO-CCSD/def2-SVP approaches; Figure S1: Vibrational modes with significant reorganization energies; Figure S2: The concerned π…π stacked dimer; Figure S3: The studied two-layered ONIOM model.
