# Supplementary material for: Insights into the Structural Modification of Selenium-Doped Derivatives with Narrowband Emissions: A Theory Study
Source: Molecules. 2024 Sep 27;29(19):4589. doi: 10.3390/molecules29194589 (PMC11477722; doi:10.3390/molecules29194589)
Supplement: Supplementary file 1 [file molecules-29-04589-s001.zip › molecules-3221660-supplementary.pdf]

Insights into the Structural Modification of Selenium-Doped Derivatives with Narrowband Emissions: A Theory Study

Qing Zhang [1], Tao Liu [1], Xin Huang [2], Kunyan Wang [1], Fangxiang Sun [1], Xin Wang [2,*] and Chunyan Lv [1,*]

1 Department of Materials Chemistry, Huzhou University, Huzhou 313000, China
2 Henan-Macquarie University Joint Centre for Biomedical Innovation, Henan Key Laboratory of Brain Targeted Bio-Nanomedicine, School of Life Sciences, Henan University, Kaifeng 475004, China
* Correspondence: wx@henu.edu.cn (X.W.); lcy@zjhu.edu.cn (C.L.)

Table S1. Contribution of each non-hydrogen atom to hole and electron of the structure-modified derivatives Cb-BSeN and PhCb-BSeN.

	Cb-BSeN		PhCb-BSeN	
Atoms	Hole (%)	Electron (%)	Hole (%)	Electron (%)
Se1	37.93	2.77	30.2	3.08
N2	4.91	0.63	7.6	0.77
C3	2.25	3.04	2.33	3.57
C4	0.41	0.58	0.1	0.78
C5	11.51	0.37	11.92	0.43
C7	2.57	4.38	2.35	4.79
C8	0.25	4.45	0.22	5.13
C10	3.88	0.52	3.53	0.9
C11	0.27	1.18	0.46	1.25
C12	11.62	0.13	12.18	0.17
C14	1.42	0.31	2.53	0.28
C15	0.99	4.38	1.59	3.5
C16	0.83	2.46	1.34	1.98
C17	0.78	3.72	0.32	4.51
C19	0.24	12.07	0.08	11.19
C21	0.7	12.86	1.05	10.79
C23	0.06	2.81	0.18	2.67
C24	1.9	3.72	2.74	2.53
C25	2.55	2.64	2.09	3.07
C26	4.39	0.57	3.03	0.8
C28	6.2	0.09	4.66	0.1
C29	0.25	1.29	0.8	0.73
C31	0.06	3.3	0.04	2.66
C33	0.25	4.01	0.3	4.97
C35	1.34	0.06	2.25	0.07
C37	1.19	0.78	2.31	0.86

B38	0.29	24.63	0.16	26.22
C39	0.02	0.05	0.01	0.02
C40	0.02	0.06	0.01	0.01
B42	0.04	0.02	0.01	0.01
B44	0.02	0.02	0.01	0.01
B46	0	0.01	0.01	0.01
B48	0.02	0.06	0.01	0.01
B50	0.01	0.02	0.01	0.01
B52	0.04	0.05	0	0.01
B54	0.03	0.06	0	0.01
B56	0.01	0.01	0	0
B58	0.03	0.04	0	0.01
B60	0.01	0.01	0.01	0.01
C62	0.03	0.01	0.01	0
C63	0.01	0.08	0	0
B65	0.05	0.21	0.01	0
B67	0.03	0.01	0.01	0
B69	0.03	-0.02	0.01	0
B71	0.05	0.14	0.02	0
B73	0.04	0.22	0.01	0
B75	0.01	0.04	0	0
B77	0.01	0.09	0	0
B79	0.01	0.03	0	0
B81	0.02	0.04	0	0
B83	0.04	0.06	0.01	0
C86	-	-	0.12	0.16
C87	-	-	0.45	0.11
C88	-	-	0.45	0.06
C89	-	-	0.02	0
C91	-	-	0.02	0.05
C93	-	-	0.52	0.03
C96	-	-	0.11	0.18
C97	-	-	0.39	0.22
C98	-	-	0.41	0.17
C99	-	-	0.02	0.02
C101	-	-	0.02	0.07
C103	-	-	0.46	0.27

Table S2. Decomposed interaction energies of the π-stacked dimers selected from the crystal structure of CzBSe at different external pressures, in which energies are in kJ/mol.

	electrostatic	repulsion	dispersion	total
0 GPa	-1.93	47.42	-126.95	-81.46
2 GPa	-1.67	100.42	-181.29	-82.54
5 GPa	-1.69	167.33	-228.76	-63.11
9 GPa	-1.65	263.44	-280.42	-18.63

Table S3. Vertical excitation energies of S_1 and T_1 (eV), the maximum wavelength of fluorescence (λ_{emi}, nm), S_1-T_1 energy gaps (ΔE_{S1T1}, eV) predicted by using B3LYP, PBE0, MN15, and M062X functionals with def2-SVP basis sets, respectively, as well as the λ_{emi} and ΔE_{S1T1} values obtained in the experiment [17,18].

		B3LYP	PBE0	MN15	M062X	Expt.
**CzBSe**	S_1	2.67	2.77	2.93	3.16	2.60
	λ_{emi}	465	448	423	393	477
	T_1	2.32	2.36	2.54	2.72	-
	ΔE_{S1T1}	0.35	0.40	0.40	0.43	0.12
**Cz-BSeN**	S_1	2.65	2.75	2.91	3.13	2.59
	λ_{emi}	467	450	427	396	479
	T_1	2.31	2.36	2.53	2.70	-
	ΔE_{S1T1}	0.34	0.39	0.38	0.43	0.15

Table S4. Calculated energy gaps between S_1 and T_1(ΔE_{ST}, eV) by the wavefunction-based STEOM-DLPNO-CCSD/def2-SVP calculations, together with energy gaps between S_1 and T_1 (ΔE_{expt}, eV) obtained in experiment.

	STEOM-DLPNO-CCSD	Expt.
**CzBSe**	0.20	0.12
**Cz-BSeN**	0.19	0.15

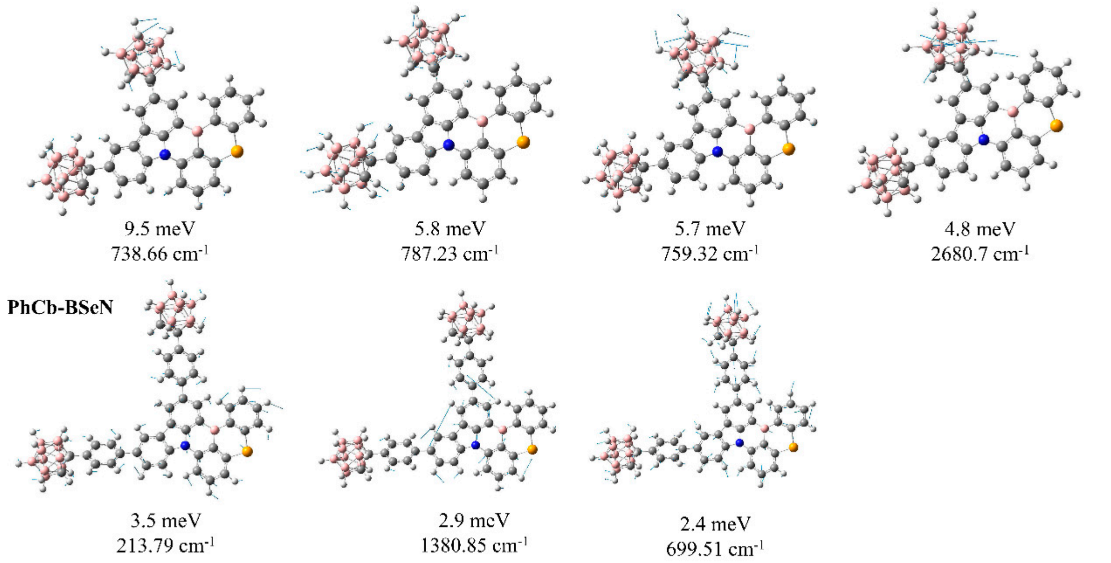

Figure S1. Vibrational modes with significant reorganization energies for Cb-BSeN and PhCb-BSeN.

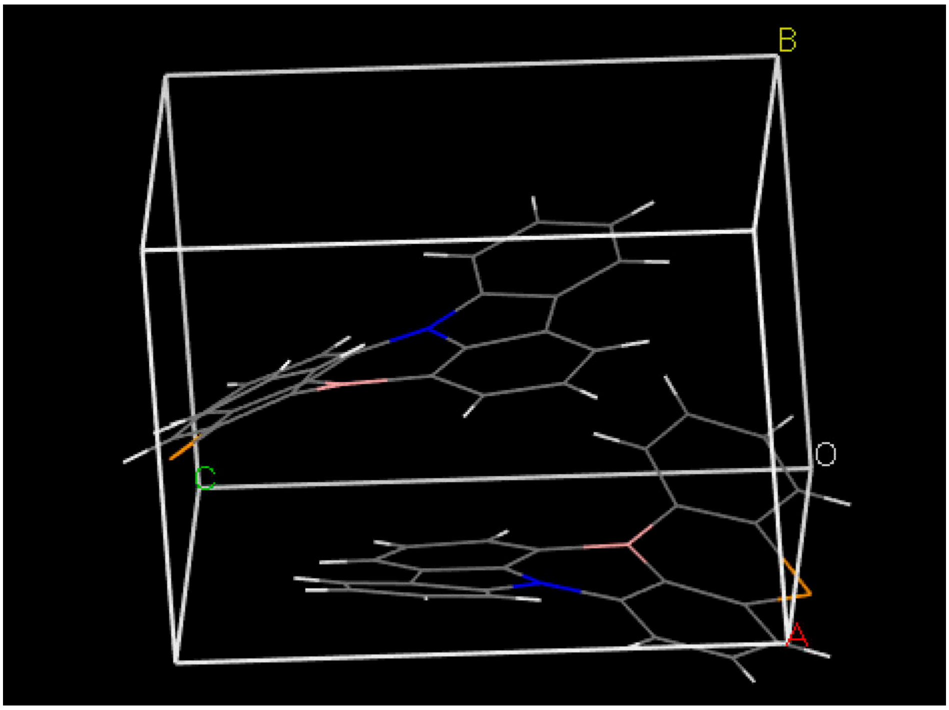

Figure S2 The π⋯π stacked dimer in the crystal structure of CzBSe.

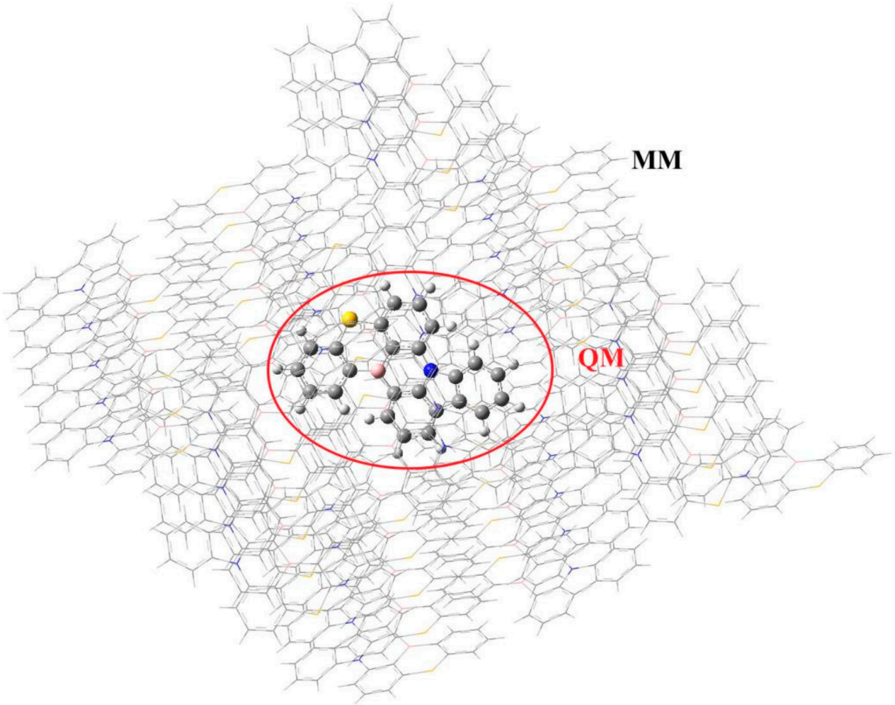

Figure S3. The two-layered ONIOM model of CzBSe in the aggregated state.
